# A structured approach to hypotheses involving continuous exposures over the life course

**DOI:** 10.1093/ije/dyw164

**Published:** 2016-07-01

**Authors:** Andrew DAC Smith, Rebecca Hardy, Jon Heron, Carol J Joinson, Debbie A Lawlor, Corrie Macdonald-Wallis, Kate Tilling

**Affiliations:** 1 School of Social and Community Medicine; 2 MRC Integrative Epidemiology Unit, University of Bristol, Bristol, UK; 3 MRC Unit for Lifelong Health and Ageing, University College London, London, UK

**Keywords:** Life course, structured approach, least angle regression (LARS), ALSPAC

## Abstract

**Background:** Epidemiologists are often interested in examining different hypotheses for how exposures measured repeatedly over the life course relate to later-life outcomes. A structured approach for selecting the hypotheses most supported by theory and observed data has been developed for binary exposures. The aim of this paper is to extend this to include continuous exposures and allow for confounding and missing data.

**Methods:** We studied two examples, the association between: (i) maternal weight during pregnancy and birthweight; and (ii) stressful family events throughout childhood and depression in adolescence. In each example we considered several plausible hypotheses including accumulation, critical periods, sensitive periods, change and effect modification. We used least angle regression to select the hypothesis that explained the most variation in the outcome, demonstrating appropriate methods for adjusting for confounders and dealing with missing data.

**Results:** The structured approach identified a combination of sensitive periods: pre-pregnancy weight, and gestational weight gain 0-20 weeks and 20-40 weeks, as the best explanation for variation in birthweight after adjusting for maternal height. A sensitive period hypothesis best explained variation in adolescent depression, with the association strengthening with the proximity of stressful family events. For each example, these models have theoretical support at least as strong as any competing hypothesis.

**Conclusions:** We have extended the structured approach to incorporate continuous exposures, confounding and missing data. This approach can be used in either an exploratory or a confirmatory setting. The interpretation, plausibility and consistency with causal assumptions should all be considered when proposing and choosing life course hypotheses.

Key Messages

- The structured approach to choosing the hypothesis, regarding an association between an exposure measured over the life course and a later outcome, that is most supported by the data can be extended to continuous exposure measurements.
- Methods for handling confounding and missing data can be incorporated into the structured approach.
- In an example investigating the association between maternal weight during pregnancy and offspring birthweight, the structured approach identified a combination of pre-pregnancy weight, weight gain during 0-20 weeks of gestation, and weight gain during 20-40 weeks of gestation, as the best explanation for variation in birthweight.
- In an example investigating the association between stressful family events and offspring birthweight, the structured approach identified a sensitive period hypothesis strengthening with the proximity of stressful family events to the point at which the outcome was measured.

## Introduction

The specific association between an exposure and outcome over the whole life course is of considerable interest in epidemiology.^1^ Different hypothesized relationships are proposed for different exposure-outcome pairs in a longitudinal setting.^2^^,^^3^ For example, competing hypotheses have been proposed for the relationship between weight across the life course and later ill health. The predictive adaptive response proposes developmental plasticity, whereby the fetus’ development is modified depending upon intrauterine environmental clues regarding available postnatal nutrition, meaning there is an interaction between birthweight (reflecting the intrauterine prediction of postnatal nutrient availability) and subsequent weight gain (reflecting the reality of postnatal nutrition).^4^^,^^5^ In contrast, the maternal capital theory proposes that the mother protects the developing fetus from current ecological conditions and health outcomes, reflecting the fit between offspring nutritional demand and maternal ability to provide this.^6^^,^^7^Box 1 describes a number of general hypotheses that, possibly in combination, could be relevant to how exposures across the life course influence later outcomes.

Box 1. Potential life course hypotheses

***Accumulation***

Under the accumulation hypothesis, the outcome has a linear association with the cumulative sum of the exposure, That is the more prolonged and/or severe the exposure, the greater the outcome. For example, a recent study examined the relationship of systolic blood pressure to future cardiovascular disease risk. The study compared short-term, intermediate and lifetime exposure, and provided support for a cumulative model that is the longer the exposure to high blood pressure across the life course, the greater the risk of cardiovascular disease.^8^

***Critical period***

The critical period hypothesis states that the outcome is only associated with the exposure at or during a critical period of the life course. For instance, exposure to thalidomide during pregnancy and while breastfeeding, but not in earlier or later periods, is associated with a risk of malformation in the offspring.

***Sensitive period***

A sensitive period hypothesis states that the outcome is associated with the exposure at all times during the life course, but the association is stronger in a particular period. For example, increased average physical activity during the whole life course may be associated with reduced risk of breast cancer, but the reduction is risk is greatest for increased physical activity during childhood and young adulthood.^9^

***Effect modification***

It may be hypothesized that the influence of an early-life factor on the outcome is modified by a later exposure or change in exposure. For example, some studies suggest an interaction between birthweight and subsequent growth, such that those who are born with lower birthweight but gain weight rapidly postnatally are at greater risk of coronary heart disease compared with other groups.^1^

***Change***

A change hypothesis states that the outcome is associated with change in the exposure. For instance, the proposed association between weight gained in the first trimester of pregnancy and offspring obesity.^10^

***Threshold***

Under a threshold hypothesis, the exposure can vary within certain limits (the thresholds) over the life course without affecting the outcome; there is only an association with exposure above or below the threshold value. For example, there is some evidence to suggest a threshold for cumulative exposure to lead in childhood, above which it affects cognitive development.^11^

There is a growing interest in a ‘structured’ approach to hypotheses relating life course exposures to later outcomes,^12^ which may allow the identification, based on observed data, of the most appropriate hypothesised model from an a priori proposed set of hypotheses. Requiring the a priori specification of plausible hypotheses prevents the development of hypotheses from observed data and encourages the specification of existing knowledge regarding the life course. The structured approach may be used to confirm an established hypothesis, or it may be used in an exploratory setting to identify which of a set of equally plausible hypotheses is most supported by observed data. We developed a structured approach for binary exposures,^13^ based on least angle regression (LARS),^14^ that demonstrated more accuracy in simulation than other methods. Our method can consider a variety of different hypotheses simultaneously, including simple ones and more compound hypotheses constructed from combinations of simple hypotheses, and allows for the calculation of unbiased *P*-values and confidence intervals.

Structured hypotheses relating life course exposures to later outcomes have been defined for binary measurements of the exposure variable, but not continuous exposure variables. Furthermore, the impact of confounding, measurement error in the exposure and missing data in the exposure and outcome have not yet been explored within the structured approach. The aim of this paper is to extend the structured approach using LARS variable selection, to hypotheses involving continuous exposures, and to take into account confounding, measurement error and missing data. The approach is illustrated using examples of the associations of gestational weight gain (GWG) with birthweight, and stressful family events with later depression.

## Methods

The structured approach can be thought of as finding an appropriate parametrization based on a priori causal assumptions concerning the exposure measurements, outcome and potential confounders. Thus a useful first step is to draw a directed acyclic graph (DAG) depicting the potential causal associations between exposure and outcome over the life course.^15^ Due to their nonparametric nature, DAGs are limited in their ability to depict many life course hypotheses such as effect modification.^16^ The causal assumptions should be used to inform the choice of a proposed set of potential hypotheses for the nature of the association between exposure and outcome over the life course. In particular, hypotheses involving exposures measured after the outcome should not be considered due to reverse causality. In the exploratory setting, this set would include any known hypothesis with biological plausibility or support in the literature, as the aim is to compare these hypotheses and also assess whether they may be working in combination. In the confirmatory setting there is one particular hypothesis of interest, which would be considered to be refuted if any competing hypothesis was, possibly in combination with the hypothesis of interest, better supported by the data. Thus in the confirmatory setting, the set of additional hypotheses should be large and varied enough to contain, in combination, all possible hypotheses permitted by the causal assumptions.

Each hypothesis is encoded into one or more variables, which are combinations of the exposure measurements. Below we detail the encoding of the hypotheses described in Box 1 into variables for use in the LARS procedure.

### 

#### Accumulation

The accumulation hypothesis can be represented by the area under the curve of exposure over time or, equivalently, the average exposure.

#### Critical period

The critical period hypothesis assumes there is an association between the exposure and outcome during one period, and no association in other periods. If the critical period is short, encompassing only one measurement occasion, the hypothesis can be represented by the exposure at that measurement occasion. A longer critical period can be represented by the average exposure over multiple measurements during that period.

#### Sensitive period

A sensitive period hypothesis is encoded through the combination of two variables: those that encode an accumulation, and critical period hypotheses. It might also be hypothesized that the ‘sensitiveness’ increases or decreases gradually over the life course, in which case the sensitive period hypotheses may be encoded by a weighted average of the exposure over the life course, with the weight increasing or decreasing with time.

#### Effect modification

An effect modification hypothesis can be encoded by the product of two variables that encode simple hypotheses. Thus, in terms of encoding, effect modification is equivalent to statistical interaction.^17^ Causal assumptions should be used to determine whether the hypothesis should be considered as effect modification or interaction.^18^

#### Change

For a continuous exposure, a simple but flexible change hypothesis may be represented by the overall change in exposure over the life course. A hypothesis in which change during a certain period is assumed to affect the outcome may be represented by the average rate of change during that period.

#### Threshold

If it is hypothesized that exceeding the threshold affects the outcome, the hypothesis would be encoded by a binary variable indicating whether the threshold has been exceeded during the life course.

Having encoded the set of potential hypotheses as a set of variables, the problem of choosing the hypothesis most supported by the observed data is translated into the problem of choosing the variable explaining the greatest proportion of variation in the outcome. As with binary exposure variables, we propose using the LARS variable selection procedure to identify these variables. Unlike stepwise variable selection procedures, LARS does not over-inflate effect size estimates during variable selection,^14^ nor bias hypothesis tests of those effect sizes.^19^ LARS will first identify the variable with the strongest association with the outcome, thus identifying the simplest hypothesis most supported by the observed data. It will then identify the combination of two variables with the strongest association with the outcome, then the combination of three variables with the strongest association and so on. This enables a choice between more complicated hypotheses (based on several variables) explaining more variation in the outcome, and less complicated but potentially more interpretable hypotheses (based on fewer variables). We suggest using an elbow plot–a plot of the proportion of outcome variation explained by the selected variable(s) (the R-squared value) against number of variables selected–to choose the number of variables. The ‘elbow’–a sharp concave bend at which increasing the number of variables does not substantially improve the fit of the selected model–is an appropriate choice of the number of variables. Alternatively, the covariance test for the lasso can test the whether the next selected variable offers an improvement in the explained proportion of the outcome variable.^19^

### Confounding

The use of a DAG to specify causal assumptions can aid the identification of potential confounders, as variables blocking back-door paths from exposure to outcome.^20^ A typical method of adjusting for such variables is to include them as covariates in the regression model. This can be achieved with existing software for LARS (see Supplementary Data, available as Supplementary Data at *IJE* online). If an elbow plot is to be used to determine the complexity of the identified hypothesis, we suggest it be truncated to show the improvement in R-squared achieved by adding additional variables subsequent to the inclusion of the confounders. In our examples we have assumed that all of the hypotheses considered have the same potential confounders; a generalization to different confounders is given in Supplementary Data, available as Supplementary Data at IJE online).

### Missing data

Individuals with missing data on one or more exposure measurements would not be included in a complete cases analysis, leading to loss of power and potential bias. We propose two existing techniques that may be combined with the LARS approach to overcome these problems. The first is imputation using chained equations.^21^ To avoid biasing the variable selection step, all combinations of exposure measurements used in variable selection, plus the outcome and any confounders, should be used in the imputation step. A multiple imputation approach may be used to obtain effect size estimates after the identification of a suitable hypothesis. However, it is recommended that variable selection be performed separately on all imputed datasets to check that the variable selection procedure is unaffected by the imputation procedure. In the special case where an exposure is measured repeatedly over the life course, multilevel models could be used to overcome the problems caused by irregularly sampled measures, or missing measures, by estimating the exposure at common time points for all participants.^22^ This has the further benefit of reducing measurement error in the exposure. Both methods are demonstrated in the following examples.

## Example 1: Gestational weight gain and birthweight

The ‘developmental overnutrition hypothesis’ suggests that greater maternal adiposity, and associated greater maternal circulating glucose and other nutrients, overfeed the developing fetus resulting in greater birthweight and adiposity throughout life.^23^^,^^24^ Under this hypothesis we might anticipate maternal early-/pre-pregnancy weight (reflecting her level of adiposity) and weight gain up to 20 weeks (when maternal fat deposition contributes more to weight gain than other constituents including fetal weight, placenta and amniotic fluid) to be related to birthweight. Alternative explanations for a relationship between GWG and offspring birthweight are: genetic variants associated with greater adiposity influence mother’s and offspring adiposity; or mother’s weight reflects her pelvic size and hence capacity to allow greater fetal growth. For both of these we might anticipate that only pre-pregnancy weight will be associated with birthweight. However, since GWG includes birthweight (the final attained weight of the fetus), later GWG where the fetus contributes more may also be important. Indeed, one might anticipate pre-pregnancy weight to interact positively with GWG, since the developing fetus of women with greater adiposity will gain more weight throughout pregnancy (under the developmental overnutrition hypothesis) and fetal weight gain will directly contribute to their greater birthweight. The Institute of Medicine (IOM) publishes recommended upper and lower thresholds for GWG according to pre-pregnancy body mass index (BMI), with the aim of reducing the implications of GWG including child obesity.^25^ Thus it could be hypothesized that moderate amounts of GWG will not have an effect on birthweight, but GWG in excess of these thresholds will.

**Figure 1.**
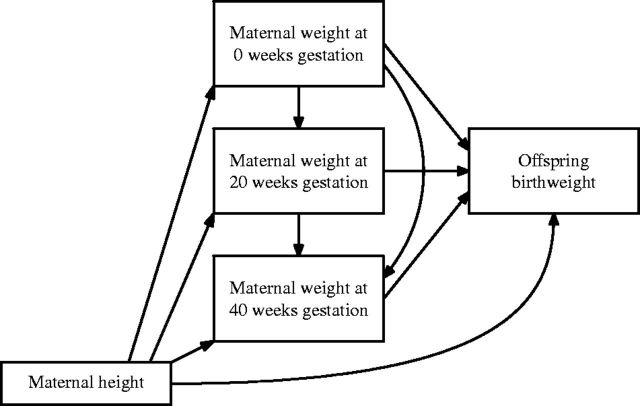
Potential DAG showing causal relationships in the association between maternal weight during pregnancy and offspring birthweight.

The set of potential hypotheses for an association between maternal weight before and during pregnancy, and offspring birthweight, are therefore: (i) pre-pregnancy critical period; (ii) pre-pregnancy critical period added to change (GWG up to 20 weeks); (iii) change (GWG from 20 to 40 weeks); (iv) effect modification between pre-pregnancy weight and total GWG; and (v) IOM threshold. Figure 1 shows a DAG depicting the potential causal relationships between the exposure and outcome measurements referred to in hypotheses (i)-(v), and also maternal height, which is a potential confounder.

### Materials, methods and preparation

The Avon Longitudinal Study of Parents and Children (ALSPAC) is a prospective population-based birth cohort study that recruited 14 541 pregnant women resident in Avon, UK, with expected dates of delivery 1 April 1991 to 31 December 1992.^26^^,^^27^ The study website contains details of all the data available through a fully searchable data dictionary [http://www.bris.ac.uk/alspac/researchers/data-access/data-dictionary]. Ethical approval for the study was obtained from the ALSPAC Ethics and Law Committee and the Local Research Ethics Committees.

Details of how GWG measurements were obtained are given in Supplementary Data (available as Supplementary Data at *IJE* online). Briefly, we used a multilevel model to obtain estimated maternal weight at 0 (i.e. pre-pregnancy), 20 and 40 weeks of gestation in 11 499 mother-offspring pairs.^28^ Such estimates overcame the issues of irregular sampling and missing data in the exposure measurements. Denoting the estimated weight of the *i*th mother at *t* weeks gestation by *x_ti_*, we encoded hypotheses (i)-(v) as follows. The critical period hypothesis (i) is encoded by the variable *C*, where *C_i_* = *x*_0__*i*_. Change from 0 to 20 weeks and from 20 to 40 weeks are encoded by Δ_1_ and Δ_2_ respectively, where Δ_1__*i*_ = *x*_20__*i*_ – *x*_0__*i*_ and Δ_2__*i*_ = *x*_40__*i*_ – *x*_20__*i*_. Total GWG is encoded by Δ_3_, where Δ_3__*i*_ = *x*_40__*i*_ – *x*_0__*i*_. Hypothesis (ii) is encoded by *C* and Δ_1_ in combination, and hypothesis (iii) is encoded by Δ_2_. The element-wise product *CΔ*_3_ encodes the interaction between pre-pregnancy weight and total GWG. Combination of this variable with *C* and Δ_3_ identifies an effect modification hypothesis (iv). LARS can suffer from low power if some variables are highly correlated,^13^ therefore we centred *C* and Δ_3_ to reduce their correlation with their product. If *U_i_* and *L_i_* denote the IOM upper and lower thresholds relevant to the *i*th mother, then the threshold hypothesis (v) can be encoded by the binary variables *T*_1_ and *T*_2_, where *T*_1__*i*_ = 1(Δ_3__*i*_ > *U_i_*) and *T*_2__*i*_ = 1(Δ_3__*i*_ < *L_i_*). We adjusted for potential confounding by maternal height using the method discussed above. Height may have a non-linear (power) relationship with weight,^29^^,^^30^ so we used LARS to identify a linear representation of height as the most appropriate power to include.

### Results

**Figure 2.**
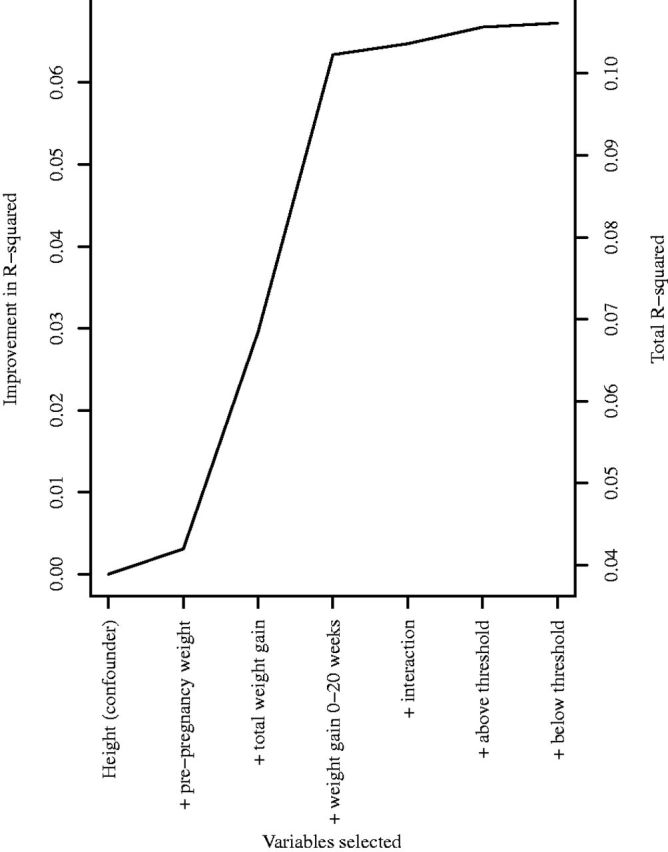
Plot of the improvement in coefficient of variation, after adjusting for confounding by maternal height, against number of variables (in addition to maternal height) selected at each stage of LARS procedure, for hypothesized association between maternal weight during pregnancy and offspring birthweight in 11 499 mother-offspring pairs.

Figure 2 shows the elbow plot of additional proportion of outcome variation explained by the selected model, after adjusting for maternal height, against number of additional variables in the selected model. The clearest elbow is at three additional variables, which are pre-pregnancy weight, total GWG and GWG between 0 and 20 weeks (R-squared value 12%). Since the combination of two GWG variables allows for two different coefficients for the two different periods 0 to 20 weeks and 20 to 40 weeks, the selected variables combine to encode hypotheses (ii) and (iii) working in combination. GWG between 0 and 20 weeks has a larger effect per kg than GWG between 20 and 40 weeks (Table 1), so 0 to 20 weeks may be thought of as the more sensitive period.

**Table 1.** Association between maternal weight in pregnancy and birthweight in 11 499 mother-offspring pairs. Adjusted for maternal height. *P*-values come from the covariance test for the lasso

	Mean (SD)	Change in birthweight (g) per kg increase (95% confidence interval)	*P*
Pre-pregnancy weight (kg)	60.7 (12.6)	10 (10, 11)	0.0001
Weight gain between 0 and 20 weeks of gestation (kg)	6.6 (3.3)	30 (26, 33)	0.0001
Weight gain between 20 and 40 weeks of gestation (kg)	9.9 (3.5)	10 (7, 13)	<0.0001

## Example 2: Stressful family events and depression

It has been suggested that exposure to multiple stressors has a greater adverse effect on mental health than exposure to single stressors,^31^ and therefore it is likely that mental health outcomes such as depression will increase in severity with increased number of stressful family events. Alternatively, there is growing evidence that exposure to adversities in early childhood are more strongly associated with subsequent mental health problems than exposure to these events in later childhood and adolescence, as specific regions of the developing brain during early childhood might be vulnerable to adverse exposures that could increase risk for depression.^32^ Family stability in the first 5 years of life may play a role in subsequent development of depression.^33^ Another potential mechanism is the ‘recency hypothesis’,^34^ in which adverse exposures have time-limited depressogenic effects, meaning proximal exposures are predicted to have stronger associations with depression compared with distal exposures. Therefore a set of potential hypotheses for an association between stressful family events in childhood, and depressive symptoms in adolescence, are: (i) accumulation; (ii) critical period from 0-5 years; (iii) sensitive period from 0-5 years; and (iv) recency.

Details of how data on stressful family events (as a discrete exposure variable) and depressive symptoms (a continuous outcome) were obtained, in 3240 female ALSPAC study children, are given in Supplementary Data, available as Supplementary Data at IJE online), as are details of how we encoded the hypotheses (i)-(iv) and a DAG showing potential causal relationships between the exposure measurements and outcome. We imputed missing data in the exposure using chained equations.

### Results

**Figure 3.**
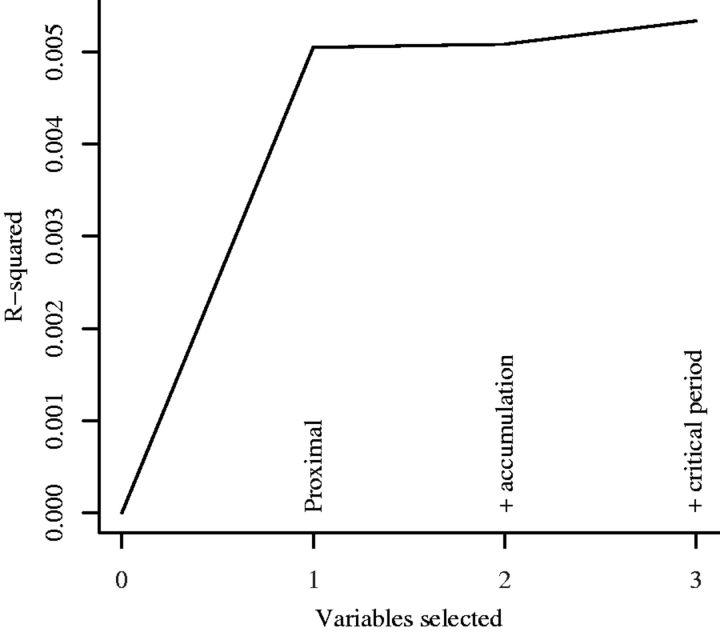
Plot of coefficient of variation against number of variables selected at each stage of LARS procedure, for hypothesized association between stressful family events (between 6 and 103 months of age) and depressive symptoms (at 14 years of age) in 3240 females.

Figure 3 shows the elbow plot for depression and stressful family events. The R-squared value for all models is less than 1%, indicating that very little of the depression score at age 14 can be attributed to stressful family events over the preceding life course. However, there is strong evidence, from the clear elbow (at one variable) on the plot and *P*-value given from the covariance test (*P* < 0.0001), that the recency hypothesis (iv) explained as much as possible of the variation in the outcome.

To test whether the observed result may have been affected by the imputation procedure, we generated a further 19 imputed datasets. In all 20 datasets, the LARS procedure selected hypothesis (iv) as the best explanation for the observed data. The results from such a model are shown in Table 2, and were obtained by averaging over all 20 imputed datasets. A single stressful family event was always associated with an increase in the depression score; however, this association increased by 0.014 [95% confidence interval (CI): 0.007, 0.022] for each additional year of age.

**Table 2.** Proximal model identified by LARS for association between stressful family events between 6 months and 103 months of age and depression score at age 14 years, in 3240 females

Age in years at event(s)	Change in depression score per event (95% confidence interval)	*P*-value from covariance test
2	0.029 (0.014, 0.045)	
4	0.059 (0.028, 0.089)	
6	0.088 (0.042, 0.134)	
8	0.117 (0.056, 0.178)	< 0.0001

## Discussion

We have demonstrated how structured hypotheses may be defined for the life course association between continuous exposure variables and a later life outcome, by extending the structured approach using LARS for variable selection beyond the current limitation to binary exposures. Additionally, we have discussed methods for handling confounding, measurement error and missing data, which previously had not been considered within the structured approach. In our GWG example, we showed a suitable adjustment for confounding within the LARS structured approach, by ensuring that LARS selected pre-pregnancy height in all models. We also combined LARS with a multilevel model in order to overcome the issues of missing data and measurement error among the exposure measurements. Finally, in our stressful family events example, we showed how the alternative method for handling missing data, imputation using chained equations, may be used in conjunction with the LARS structured approach and was robust to random changes between imputed datasets.

Other statistical methods applicable to the structured approach exist, such as comparing hypothesized models against a saturated model using an F-test.^12^ This cannot be extended to continuous exposures, as the required saturated model would have as many parameters as study participants. Another method combined the Akaike Information Criterion (AIC) with an F-test.^35^ The AIC could be used alone for structured hypotheses involving continuous exposures. However, this method lacks statistical power when compared with LARS.^13^ Other variable selection procedures, such as stepwise regression, the grouped lasso or the elastic net, could be applied in place of LARS. However, stepwise regression would introduce biases that LARS is known to overcome,^19^ and we have previously discussed how the grouped lasso and elastic net methods would result in the identification of needlessly complicated hypotheses.^13^

As with other methods for the structured approach, strong correlation between the exposure measurements may decrease statistical power.^13^ Indeed, if we did not centre terms in our first example, the strong correlation with the interaction variable would result in identifying an effect modification hypothesis instead of the additive one reported in Table 1. In all methods for the structured approach, it is unclear how much the selection of hypotheses is affected by measurement error in the exposure. We have assumed that the exposure is always measured with the same level of precision, as variable selection may be affected by the variance of the exposures. Although not shown here, LARS can also be used to test different hypothesized life course associations in different exposure variables. These too would have to be assumed to have the same level of measurement error. In Supplementary Data, available as Supplementary Data at IJE online). we give details of a method for adjusting for confounders when different hypothesized life course associations have different potential confounders. However, this method does not allow for the potential combination of more than one hypothesized association with different potential confounders, as may be encountered if effect modification is considered.^37^

In our first example, pre-pregnancy weight and GWG were positively associated with birthweight. Thus the observed data provide some support for the developmental overnutrition hypothesis, as women with greater pre-pregnancy weight might on average have greater fat mass, which across pregnancy results in overnutrition of their infant. We note that GWG combines a number of phenotypes (maternal fat deposition, maternal volume expansion, fetal growth, placenta, amniotic fluid) and our findings might differ with better exposure measurements. For example, having ultrasound scan measures of maternal and fat deposition during pregnancy might better define the influence of maternal adiposity on offspring adiposity.

The results of our second example supported the recency hypothesis^34^ in which proximal, rather than distal, exposure to stressful life events was more strongly associated with depression. This does not mean that distal exposures do not influence susceptibility to depression. According to the hopelessness theory of depression, distal contributors (e.g. attributional style) influence causal attributions related to proximal life events.^36^ Distal contributors, therefore, could make individuals vulnerable to experiencing depression when faced with proximal stressful life events. Our analysis assumed a linear association between stressful life events and depression, which would need to be investigated further, as would the definition of a stressful life event and whether some could be more influential than others. We did not investigate nonlinear relationships in either of the examples above, preferring to treat nonlinear associations as special cases of hypotheses that could, if desired, be investigated by encoding additional variables, for instance powers or fractional polynomials.

The structured approach will fail to identify the underlying association if it is not included in the set of hypotheses proposed a priori. One of the advantages of the LARS approach is that variables from different hypotheses can combine to form new hypotheses not originally proposed. Thus the correct underlying association may be identified in spite of not being included a priori. This is likely to occur when the set of potential hypotheses is general enough to include, in combination, all possible associations permitted by the causal assumptions. The structured approach may be used to assess the extent to which hypotheses concerning the association between continuous exposure measurements and an outcome are supported by observed data.

## Funding

This work was supported by the Medical Research Council [grant number G1000726]. A.S., C.M-W., D.L. and K.T. work in a unit that receives funding from the Medical Research Council [MC_UU_12013/5 and MC_UU_12013/9]. D.L. receives support from a National Institute for Health Research Senior Investigator awards [NF-SI-0611-10196]. C.M-W. is funded by a Medical Research Council research fellowship [MR/J011932/1]. R.H. is supported by the Medical Research Council [MC_UU_12019/2]. The UK Medical Research Council and the Wellcome Trust [102215/2/13/2] and the University of Bristol provide core support for ALSPAC. Aspects of this research were funded by the Economic and Social Research Council [RES-000-22-2509]. This publication is the work of the authors, and A.S. and K.T. will serve as guarantors for the contents of this paper.

## Supplementary Material

Supplementary Data

Supplementary Data
