# Supplementary material for: A structured approach to hypotheses involving continuous exposures over the life course
Source: Int J Epidemiol. 2016 Jul 1;45(4):1271–9. doi: 10.1093/ije/dyw164 (PMC5841633; doi:10.1093/ije/dyw164)
Supplement: Supplementary Data [file dyw164_supplementary_data.zip › ije-2015-04-0512-File005.docx]

# Supplementary Material

## Details of how gestational weight gain (GWG) measurements were obtained

The majority (13 706) of women in ALSPAC provided assent for their obstetric records to be abstracted. Six trained research midwives abstracted data from obstetric medical records, including every entered measurement of weight (measured at all antenatal clinic visits in the UK at the time of data collection) and the corresponding gestational age and date at the time of the weight measurement. Multilevel models with measurement occasions nested within women, were used to model the pattern of GWG for all women.^1^ A multilevel linear spline model with two knots identified three distinct periods of approximately linear weight gain: 4-18weeks, 18-28 weeks and 28+ weeks. The individual-level residuals from this model were used to predict maternal weight at different gestational ages. We excluded 189 multiple pregnancies, pregnancies for which offspring sex was not recorded (5, none of which were live births), and 329 women with no weight measurements recorded during pregnancy or no dates at weight measurements. We further restricted our analysis to 11 499 mother-offspring pairs with complete data on birthweight and pre-pregnancy maternal BMI.

## Details of how data on stressful family events and depressive symptoms were obtained

Since the birth of the ALSPAC study children, the main carers have completed regular questionnaires about their child. On seven occasions (18, 30, 42, 57, 69, 81 and 103 months) these questionnaires contained a list of potentially stressful life events which the child may have experienced.^2^ We selected a core set of five events, repeated in all questionnaires, relating to disruptions to family stability (separation from mother, separation from father, obtaining a new parent, experiencing a change of main-carer, and moving house). We used an ordinal exposure scale of 0 to 5 to indicate how many of these events were reported at each time point. In this analysis, we chose to focus on females due to the well-established increase in depressive symptoms in females compared with males during adolescence.^3^ To overcome missing and irregularly sampled outcome data, we used repeated measures of the Short Moods and Feelings Questionnaire to estimate a latent growth model describing changes in the level of depressive symptoms from 13 to 18 years,^4,5^ using the estimated latent trait of depressive symptoms at age 14 years as the outcome in this example. Data from at least one measurement occasion was available for 3240 females.

## Details of encoding hypotheses for stressful family events and depression

Where $x_{ij}$ is the number of stressful family events for participant *i* at age $t_{ij}$.

| Hypothesis |  | Encoding |
| --- | --- | --- |
| (i), (iii) | Accumulation | $\sum_{j=1}^{7} x_{ij}$ |
| (ii), (iii) | Critical period (0-5 years) | $\sum_{j=1}^{7} I_{\left[ 0,5 \right]}\left( t_{ij} \right)x_{ij}$ |
| (iv) | Sensitive period proximal to outcome measurement | $\sum_{j=1}^{7} t_{ij}x_{ij}$ |

## Potential directed acyclic graph (DAG) showing causal relationships in the association between stressful life events (between 6 and 103 months of age) and depressive symptoms (at 14 years of age)


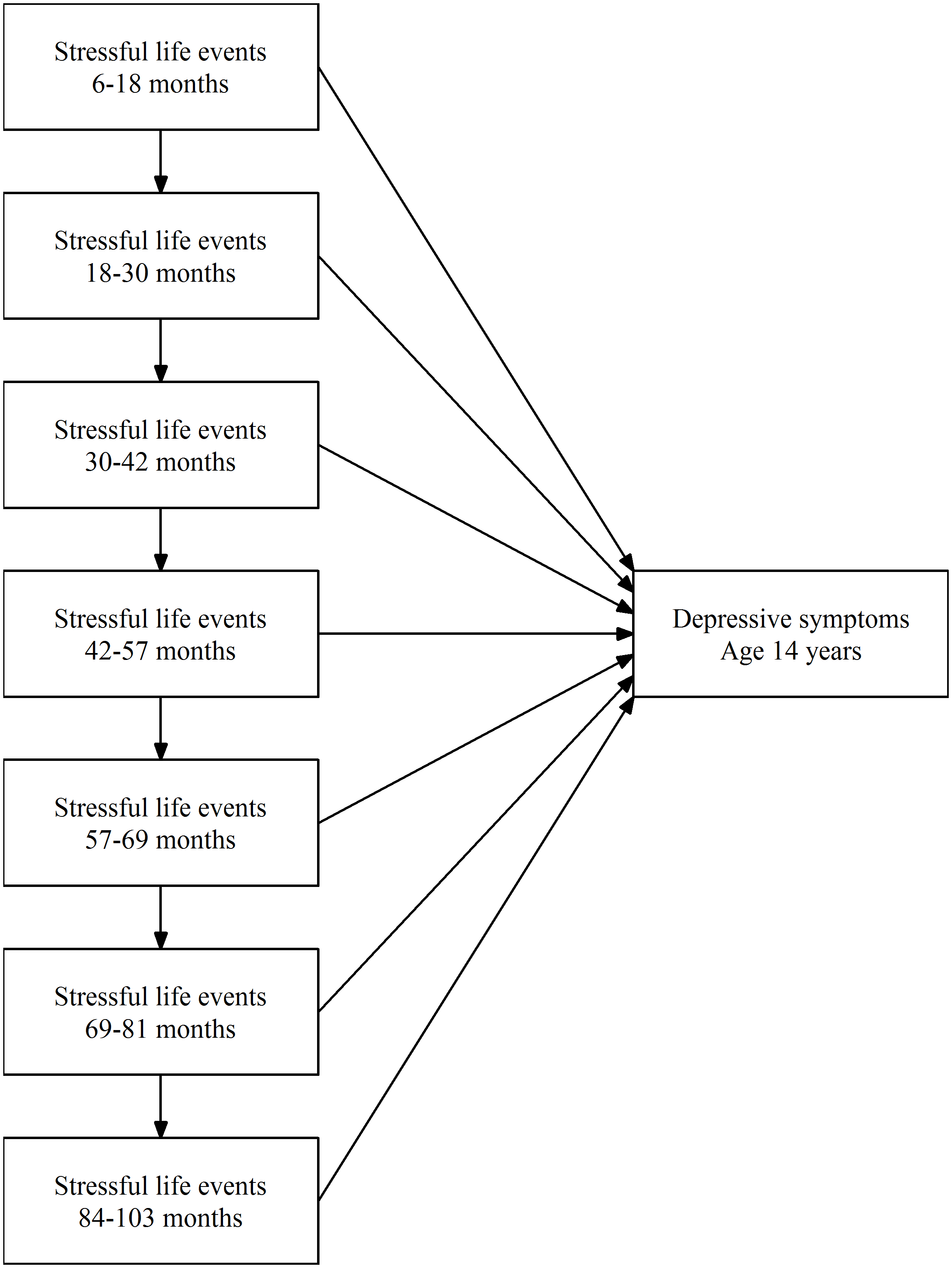


Conceptually, this DAG would include another 15 arrows joining all possible pairs of exposure measurements. However, these have been omitted for clarity.

## Software details

Here we give a description of, and code for, a method for adjusting for potential confounders that can be used with existing software in the R statistical language. We demonstrate with a simple simulated example in which the true underlying model is a critical period at the third exposure measurement, and there is a single covariate that acts as a confounder.

### R code for setup

library(lars)

# Choose a sample size

n <- 400

# Simulate a covariate

set.seed(1234)

covariate <- rnorm(n)

# Simulate exposure variables

x1 <- covariate + rnorm(n)

x2 <- x1 + rnorm(n)

x3 <- x2 + 2*rnorm(n)

# Simulate an outcome, e.g. critical period model with confounding

y <- 2*covariate + x3 + 3*rnorm(n)

# Encode some typical hypotheses

critical_1 <- x1

critical_2 <- x2

critical_3 <- x3

accumulation <- (x1+x2+x3)/3

change_1 <- x2-x1

change_2 <- x3-x2

change_3 <- x3-x1

### R code for LARS without adjusting for confounders

# Apply LARS, treating the covariate like all the other key variables

X_hypos <- cbind(covariate, critical_1, critical_2, critical_3, accumulation,

change_1, change_2, change_3)

lasso <- lars(X_hypos, y)

attributes(lasso$actions[[1]])$name #1st variable selected

attributes(lasso$actions[[2]])$name #2nd variable selected

The first variable selected encodes an accumulation hypothesis, which is not the correct selection for this simulation. The second variable selected is the covariate. As the covariate is selected after the first key variable, the selection of the key variable is made without adjusting for the covariate. As a result, the hypothesis chosen is incorrect.

### R code for adjusting for confounders

To ensure that the selection of key variables takes place with adjustment for confounding, we need to include the confounder variable in all steps of the LARS procedure. There is no automatic feature for this purpose in the lars R package.^6^ However, it can be caused to happen by altering the way the algorithm used by this package calculates correlations. The first variable selected by this algorithm will be that with the largest correlation with the outcome, based on an internal calculation of the correlation. This calculation assumes all variables have a Euclidean norm of 1,^7^ so the LARS routine initially normalizes all variables to ensure this. If the norm of the variable is greater than 1, the internal calculation of the correlation will be artificially inflated (without altering the true correlation between any of the variables. We overrode the initial normalization, manually normalized all variables and multiplied the (normalized) confounding variable by a large factor. As a consequence the confounder was selected before any other variables and included in all models. We found that the particular choice of multiplying factor did not affect the variable selection procedure, consistent with other numerical studies involving the lasso,^8^ and a factor of 1000 seems sufficient.

# Normalize covariate and key variables

col_mean <- apply(X_hypos, 2, mean)

X_centered <- X_hypos - rep(col_mean, rep(n, 8)) #subtract mean

col_sss <- apply(X_centered, 2, function(x) sqrt(sum(x^2)))

X_normed <- X_centered / rep(col_sss, rep(n, 8)) #divide by sqrt sum squares

# Multiply covariate by 1000

X_normed[,1] <- 1000*X_normed[,1]

# Apply LARS, with user-specified normalization

lasso <- lars(X_normed, y, normalize=FALSE)

attributes(lasso$actions[[1]])$name #1st variable selected

attributes(lasso$actions[[2]])$name #2nd variable selected

The covariate is the first variable selected, and this is guaranteed. Therefore selection of the next variable (the first key variable that will be used to identify a hypothesis) is made after adjusting for confounding. The second variable selected encodes, correctly, the critical period hypothesis.

### R code for producing an elbow plot

# Collect information from LARS

last.action <- length(lasso$actions)

additions <- character(last.action)

variables <- numeric(last.action)

variables[1] <- 1

current_selection <- lasso$beta["1",] != 0

for(action in 2:last.action) {

new_selection <- lasso$beta[as.character(action),] != 0

variables[action] <- sum(new_selection)

if(variables[action] > variables[action-1]) {

additions[action] <-

dimnames(X_hypos)[[2]][new_selection != current_selection]

}

current_selection <- new_selection

}

# Elbow plot

par(mar=c(5,4,4,5)+0.1)

plot(variables-variables[1], lasso$R2[-1]-lasso$R2[2], type='l',

xlab="Additional variables selected", ylab="Improvement in R-squared")

par(new=TRUE)

plot(variables-variables[1], lasso$R2[-1], type='n',

xaxt='n', yaxt='n', xlab="", ylab="")

axis(4)

mtext("Total R-squared", side=4, line=3)

text(variables-variables[1],lasso$R2[2], labels=additions, srt=90, adj=0)

### R code for verifying selection after adjusting for confounders

The above R code effectively uses the lars R package to remove the lasso penalty on the covariate, by using the numerical technique of multiplying by a large factor. An alternative method can be found within the glmnet R package.^9^ This can be used to verify the key variables selected after adjusting for confounders, but is not compatible with the covariance test for the lasso,^10,11^ so cannot be used for later calculation of p-values.

library(glmnet)

check1 <- glmnet(X_hypos, y, alpha=1, penalty.factor=c(0,rep(1,7)))

attributes(which(check1$beta[,1] != 0))$names #1st variable selected

attributes(which(check1$beta[,2] != 0))$names #1st and 2nd variables selected

Using this approach, the first variable selected is guaranteed to be the covariate. The next variable selected is the same as that selected using LARS with the multiplying factor.

A further alternative is to regress the variables on the covariate, and use the residuals in the LARS selection procedure. This too can be used to verify the key variables selected after adjusting for confounding, but does not show the initial R-squared value in the elbow plot (the amount of variation in the outcome explained by the confounder).

X_residual <- lm(X_hypos ~ covariate)$residual[,2:8]

check2 <- lars(X_residual, y)

attributes(check2$actions[[1]])$name #1st variable selected

The first variable selected, with the strongest association with the residuals, is the same as that selected above.

This final method can also be used if the different hypothesised relationships have different potential confounders. For example, consider a covariate that may have a causal association with the exposure measurement in adulthood but not in childhood. In this case, it would be necessary to adjust for potential confounding in an adult critical period model, but not in a childhood critical period model. Each variable that encodes a hypothesis could be individually regressed on the covariate(s) relevant to the particular hypothesis that it encodes, and the residuals used in the LARS selection procedure.

# References

1. Fraser A, Tilling K, Macdonald-Wallis C, et al. Association of maternal weight gain in pregnancy with offspring obesity and metabolic and vascular traits in childhood. Circulation 2010; 121: 2557-64.
2. Coddington RD. The significance of life events as etiological factors in the diseases of children. J Psychosom Res 1972; 16: 7-18.
3. Hankin BL, Abramson LY, Moffitt TE, Silva PA, McGee R, Angell KE. Development of depression from preadolescence to young adulthood: emerging gender differences in a 10-year longitudinal study. J Abnorm Psychol 1998; 107: 128-40.
4. Angold A, Costello EJ, Messer SC. Development of a short questionnaire for use in epidemiological studies of depression in children and adolescents. Int J Methods Psychiatr Res 1995; 5: 237-49.
5. Edwards AC, Joinson C, Dick DM, et al. The association between depressive symptoms from early to late adolescence and later us and harmful use of alcohol. Eur Child Adolesc Psychiatry 2014; 23: 1219-30.
6. Hastie T, Efron B. lars: Least angle regression, lasso and forward stagewise. R package version 1.2. (2013)
7. Efron B, Hastie T, Johnstone I, Tibshirani R. Least angle regression. Ann Stat 2004; 32: 407-99.
8. Smith ADAC. Quadratic programming and penalized regression. Commun Stat A-Theory 2013; 42: 1363-72.
9. Friedman J, Hastie T, Tibshirani R. Regularization paths for generliazed linear models via coordinate descent. J Stat Soft 2010; 33: 1-22.
10. Lockhart R, Taylor J, Tibshirani R, Tibshirani R. A significance test for the lasso. Ann Stat 2014; 42: 413-68.
11. Lockhart R, Taylor J, Tibshirani R, Tibshirani R. covTest: Computes covariance test for adaptive linear modelling. R package version 1.02. (2013)
