# Supplementary material for: A structured approach to hypotheses involving continuous exposures over the life course
Source: Int J Epidemiol. 2016 Jul 1;45(4):1271–9. doi: 10.1093/ije/dyw164 (PMC5841633; doi:10.1093/ije/dyw164)
Supplement: Supplementary Data [file supp_45_4_1271__index.html]

A structured approach to hypotheses involving continuous exposures over the life course — Supplementary Data 

# A structured approach to hypotheses involving continuous exposures over the life course

## Supplementary Data

files

- Supplementary Data - docx file
